# Stress Response of Aphid Population Under Combined Stress of Cadmium and Lead and Its Effects on Development of *Harmonia axyridis*

**DOI:** 10.3390/ijms252011145

**Published:** 2024-10-17

**Authors:** Shasha Wang, Qimei Li, Yan Li, Sijing Wan, Zhenjuan Yin, Shan Zhao, Xiaoyan Dai, Ruijuan Wang, Shigui Wang, Yifan Zhai, Xiaoling Tan, Bin Tang

**Affiliations:** 1 College of Life and Environmental Sciences, Hangzhou Normal University, Hangzhou 311121, China; wangshasha1997@163.com (S.W.); 17307849874@163.com (Q.L.); 17376552538@163.com (Y.L.); wsjw9898@163.com (S.W.); sgwang@hznu.edu.cn (S.W.); 2 Institute of Plant Protection, Shandong Academy of Agricultural Sciences, Jinan 250100, China; yinzhenjuan1220@126.com (Z.Y.); zhaoshan0328@163.com (S.Z.); 15169087554@163.com (X.D.); wangruijuan1020@126.com (R.W.); zyifan@tom.com (Y.Z.); 3 Key Laboratory of Natural Enemies Insects, Ministry of Agriculture and Rural Affairs, Jinan 250100, China; 4 State Key Laboratory for Biology of Plant Diseases and Insect Pests, Institute of Plant Protection, Chinese Academy of Agricultural Sciences, Beijing 100193, China; 5 Zhongyuan Research Center, Chinese Academy of Agricultural Sciences, Xinxiang 453500, China

**Keywords:** heavy metal stress, aphid, natural enemy, food chain, bioaccumulation, trehalose metabolism

## Abstract

Heavy metal pollution is a serious global environmental issue. It threatens human and ecological health. Heavy metals can accumulate in the soil over extended periods and inevitably transfer through the food chain to herbivorous insects and their natural enemies, leading to various adverse effects. This study aimed to investigate the stress responses and biochemical metabolic changes of aphids and one of aphids’ predators, ladybugs, under cadmium (Cd) and lead (Pb) stress by constructing a food chain of *Vicia faba* L., *Megoura crassicauda*, and *Harmonia axyridis*. The results showed that aphids and ladybugs had a notable accumulation of Cd^2+^ and Pb^2+^. Insects can adapt to heavy metal stress by regulating their energy metabolism pathways. Glycogen content in the first filial generation (F1) aphids decreased significantly, glucose content in the second filial generation (F2) to the fourth filial generation (F4) adult aphids significantly increased, and trehalose content in the F1 adult aphids increased significantly. Moreover, the relative expression levels of trehalase (*TRE*) and trehalose-6-phosphate synthase (*TPS*) in the F1 adult aphids were significantly higher than those in the control group, and the expression levels of *TPS* genes in the second filial generation to the fifth filial generation (F2 to F5) aphids decreased, suggesting that insects can resist heavy metal stress by regulating trehalose metabolism. The fertility of female aphids in all treatment groups was reduced compared to the control group. Additionally, the relative expression level of vitellogenin (*Vg*) was down-regulated in all aphid generations except the F1 aphids. There was interaction between heavy metal concentration and aphid generation, and it significantly affected the number of aphids’ offspring and the expression of the aphid *Vg* gene. The developmental duration of the ladybugs from the second to fourth instars was prolonged, and the weight decreased significantly from the prepupa to the adult stages. These results contribute to understanding the effects of Cd^2+^–Pb^2+^ accumulation on phytophagous insects and higher trophic levels’ natural enemies, laying the foundation for protecting natural enemies and maintaining ecosystem stability.

## 1. Introduction

Heavy metal pollution represents a critical concern in contemporary environmental discourse. Anthropogenic activities such as mining, metallurgical processes, and the irrigation of farmlands with sewage contribute significantly to elevated concentrations of heavy metals in soil, subsequently leading to their accumulation in food crops [1,2]. The national soil pollution survey in China indicated that heavy metals are the primary pollutants in contaminated soils, with high levels of cadmium (Cd), nickel (Ni), arsenic (As), copper (Cu), mercury (Hg), lead (Pb), chromium (Cr), and other heavy metals [3]. Due to their inherent traits of persistence and profound toxicity, heavy metals pose a severe threat to both ecosystems and human health [4,5].

When heavy metals accumulate in the soil due to anthropogenic activities, the electrochemical gradient of metal ions between the plasma membrane of plant root cells and the soil facilitates the transport of these ions from roots to other plant parts, eventually leading to their accumulation throughout the entire plant [6]. Herbivorous insects play pivotal roles in the transfer and accumulation of heavy metals [7]. Studies have demonstrated that phytophagous insect populations experience stress from heavy metals, resulting in impaired growth and development [8,9,10]. Furthermore, natural enemies indirectly suffer from heavy metal pollution as they parasitize or prey upon herbivorous insects, which hampers their own growth, development, and predatory efficiency [11,12]. These detrimental effects pose significant challenges to integrated pest management. Thus, it is crucial to investigate the potential threat posed by heavy metals to producers, consumers, and higher trophic levels through the food chain.

Cd exhibits a markedly higher mobility in soil than other heavy metals, rendering it highly susceptible to uptake by plants and subsequent transfer through the food chain to animal bodies, thereby amplifying the risk of human exposure to Cd [13,14]. Pb is one of the most prevalent heavy metals in agricultural areas and is highly phytotoxic [15]. It is readily absorbed by plants and animals and subsequently passes through the food chain into the human body. The significant accumulation of Pb in living organisms can result in the chronic poisoning of mammals and, in severe cases, even death [16,17]. Yang et al. evaluated the health analysis of five heavy metals, Cu, Cd, As, Hg, and Pb, on two medicinal insects of *Mylabris* through the soil–plant–insect system and found that the heavy metals with a higher content in the insects were Cu (48.65 and 27.82 mg/kg) and Pb (4.33 and 1.07 mg/kg), indicating that these two elements are more easily absorbed by plants and insects [18]. The majority of current research examines the impact of individual metal elements on insect growth and development [19,20,21]. Nevertheless, there is a paucity of research investigating the effects of compound heavy metal stress on insects. It is therefore imperative to investigate the impact of exposure to two or more heavy metals on insect development and the stress response mechanisms that insects employ in response to such exposure. Furthermore, examining the impact of heavy metals on organisms across various trophic levels via food chain transmission is a more effective approach for elucidating the enrichment and transmission patterns of heavy metals in plant-feeding herbivorous insects compared to a direct addition to diets or injection into insects. While some studies have explored the effects of heavy metals on organisms via soil–plant–phytophagous insect–predator food chains [22,23,24], the majority have focused on single-metal stress, thereby neglecting research from the perspective of combined stress. Consequently, it is of paramount importance to investigate the physiological and biochemical impacts of composite heavy metal stress on insects within food chain models.

To investigate the impact of two heavy metal elements on the toxicity to herbivorous insects and natural enemies through food chain transmission, based on prior studies [25,26], different concentrations of cadmium and lead were applied to soil to simulate heavy metal pollution. The accumulation levels of Cd and Pb in *Megoura crassicauda* (Hemiptera: Aphididae) and *Harmonia axyridis* (Coleoptera: Coccinellidae) as well as their effects on growth, development, metabolic mechanisms, and reproduction were assessed. This not only provides a theoretical basis and experimental reference for further exploring the molecular mechanisms of heavy metal transfer and accumulation in the food chain but also provides a theoretical basis for optimizing the biological control of pests in heavy metal contaminated areas.

## 2. Results

### 2.1. Cd, Pb Accumulation in M. crassicauda and Pupa of H. axyridis

**Figure 1:**
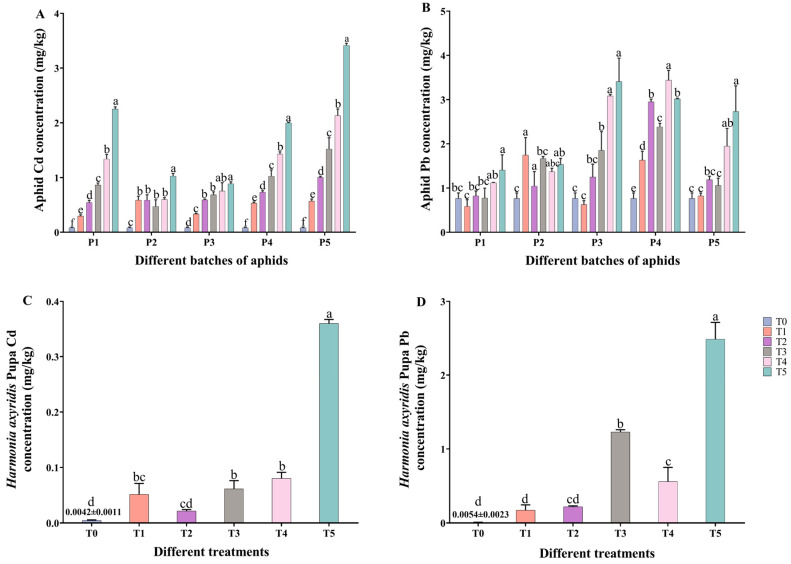
Cadmium (Cd) and lead (Pb) accumulation in *Megoura crassicauda* and pupa of *Harmonia axyridis*. (**A**) Cd concentration in different batch of aphids. (**B**) Pb concentration in different batch of aphids. (**C**) Cd concentration in the pupa of *H. axyridis*. (**D**) Pb concentration in the pupa of *H. axyridis*. Configured solutions of Cd^2+^ with concentrations of 3.125, 6.25, 12.5, 25, and 50 mg/L and Pb^2+^ with concentrations of 12.5, 25, 50, 100, and 200 mg/L were mixed in a 1:1 ratio. These mixtures were labeled as groups T1, T2, T3, T4, and T5, respectively (T0: tap water treatment, control group, T1: 3.125 + 12.5 mg/L, T2: 6.25 + 25 mg/L, T3: 12.5 + 50 mg/L, T4: 25 + 100 mg/L, T5: 50 + 200 mg/L). Results shown are means (±SE) of three replicates (n = 3). The differences in cadmium and lead concentrations in aphids and ladybug pupae among different treatments were analyzed by Tukey’s test of one-way ANOVA Different lowercase letters indicate significant differences at the *p* < 0.05 level.

The cadmium and lead concentration in aphids of the P1–P5 groups was significantly higher than that in the control group (Figure 1A,B). At the same time, the cadmium-and-lead concentration in the pupae of *H. axyridis* of the T1–T5 groups was significantly higher than that in the control group (*p* < 0.05) (Figure 1C,D). Interestingly, the concentrations of cadmium in aphids are positively correlated with the treatment level (Figure 1A). Specifically, in the first and third batches of aphids, the lead content in the T1 treatment group was lower than that in the T0 group (P1: *F*_5, 12_ = 940.768; P3: *F*_5, 12_ = 56.431; *p* < 0.001 for ANOVA), whereas in the second and fourth batches, it was significantly higher (P2: *F*_5, 12_ = 49.616; P4: *F*_5, 12_ = 503.775; *p* < 0.001) (Figure 1B). The concentrations of cadmium and lead in *H. axyridis* pupae treated with the cadmium–lead mixed solution were markedly elevated compared to those in the T0 group (Cd^2+^: *F*_5, 12_ = 400.481; Pb^2+^: *F*_5, 12_ = 168.647; *p* < 0.001). These findings underscore the transfer of heavy metals through the food chain, leading to accumulation in aphids and ladybugs subsequent to plant treatment.

**Figure 2:**
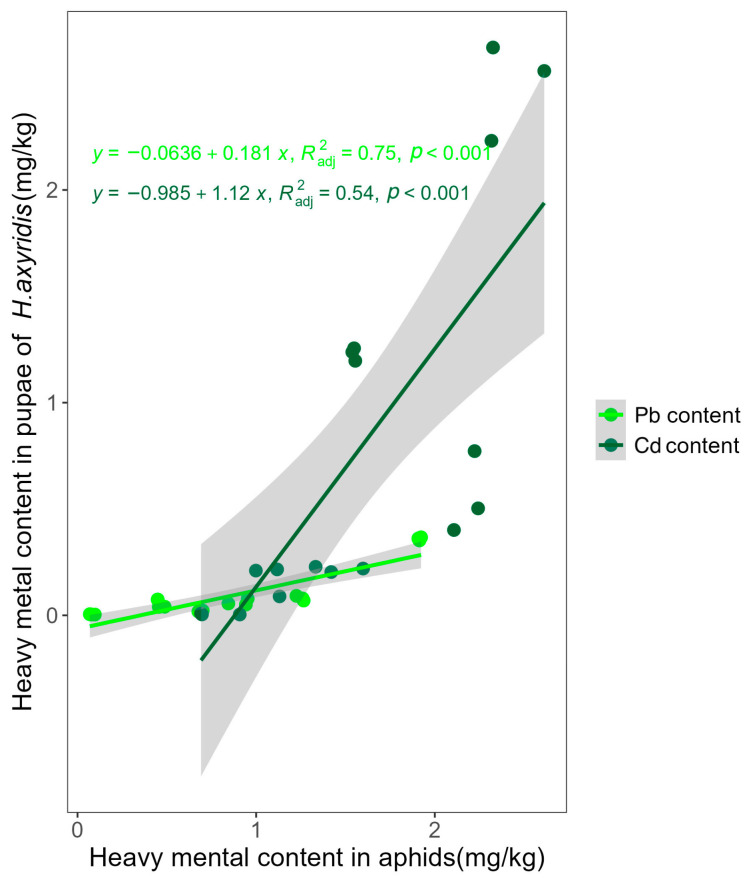
Regression between different Cd–Pb content in aphids and the heavy metal content in pupae of *Harmonia axyridis*. The gray part represents the 95% confidence interval.

According to the results of linear regression analysis, the contents of cadmium (*R*^2^_adj_ = 0.75, *p* < 0.001) and lead (*R*^2^_adj_ = 0.54, *p* < 0.001) in the pupae of *H. axyridis* were positively correlated with the contents of heavy metals in aphids (Figure 2). This finding indicates that the concentration of heavy metals in host insects directly influenced the enrichment of these metals in their natural enemies.

### 2.2. Effect of Cd–Pb Stress on Carbohydrate Contents in Five Generations of Aphids

**Figure 3:**
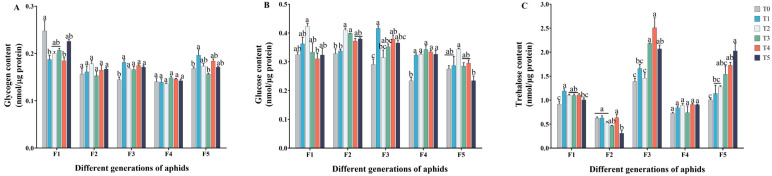
Changes in carbohydrate content in different generations of aphids after Cd–Pb combined stress. (**A**) represents the change in glycogen content during stress, (**B**) represents the change in glucose content during stress, (**C**) represents the change in trehalose content during stress. Configured solutions of Cd^2+^ with concentrations of 3.125, 6.25, 12.5, 25, and 50 mg/L and Pb^2+^ with concentrations of 12.5, 25, 50, 100, and 200 mg/L were mixed in a 1:1 ratio. These mixtures were labeled as groups T1, T2, T3, T4, and T5, respectively (T0: tap water treatment, control group, T1: 3.125 + 12.5 mg/L, T2: 6.25 + 25 mg/L, T3: 12.5 + 50 mg/L, T4: 25 + 100 mg/L, T5: 50 + 200 mg/L). Results shown are means (±SE) (n = 3). Tukey’s test of one-way ANOVA was used to analyze the differences in the carbohydrate content among different groups of the same generation. Different lowercase letters indicate significant differences at the *p* < 0.05 level.

In the first filial generation (F1) aphids, the glycogen content of the aphids in the T1 (*p* = 0.009) and T4 (*p* = 0.007) groups significantly decreased compared to the T0 group. The glycogen content of aphids in the third filial generation (F3) aphids was significantly increased compared to the control group (*F*_5, 12_ = 8.002; *p* = 0.002). However, there was no significant difference in the content of the second filial generation (F2) and the fourth filial generation (F4) aphids fed cadmium-and-lead-contaminated broad beans compared to the control group (F2: *F*_5, 12_ = 0.896; *p* = 0.514; F4: *F*_5, 12_ = 0.886; *p* = 0.520) (Figure 3A). The glucose content of the F4 aphids was significantly higher compared to the control group (*F*_5, 12_ = 17.448; *p* < 0.001) (Figure 3B). In the T5 group, the trehalose content of the F2 aphids was significantly lower compared to the control group (*p* = 0.003). For the F4 aphids, the trehalose content was not significantly different compared to the control group (*F*_5, 12_ = 1.769; *p* = 0.194) (Figure 3C). These findings highlight the impact of heavy metal treatment on the glycogen, glucose, and trehalose contents of aphids across five successive generations, revealing varying degrees of influence over time.

### 2.3. Effect of Cd–Pb Stress on Trehalose Metabolism in Five Generations of Aphids

**Figure 4:**
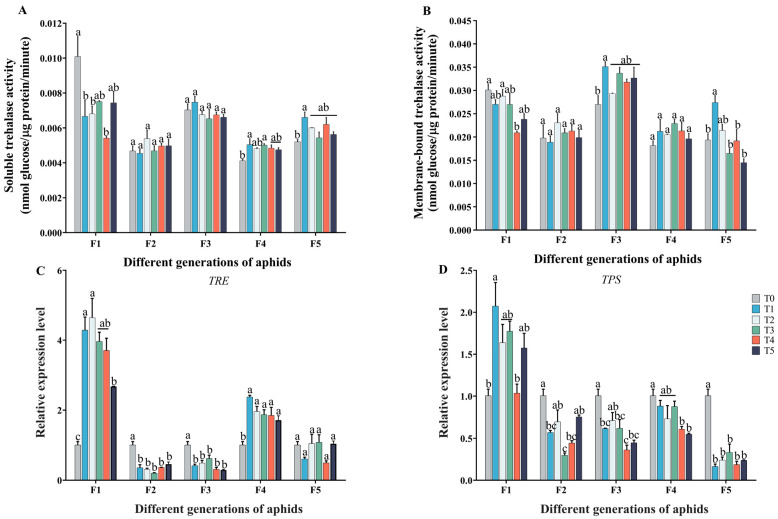
Effect of Cd–Pb stress on trehalose metabolism in five generations of aphids. (**A**) Change in soluble trehalase activity during Cd–Pb combined stress, (**B**) change in membrane-bound trehalase activity during Cd–Pb combined stress, (**C**) The expression level of trehalase (*TRE*)gene in the adult aphids, (**D**) The expression level of Trehalose-6-phosphate synthase (*TPS*) gene in the adult aphids Configured solutions of Cd^2+^ with concentrations of 3.125, 6.25, 12.5, 25, and 50 mg/L and Pb^2+^ with concentrations of 12.5, 25, 50, 100, and 200 mg/L were mixed in a 1:1 ratio. These mixtures were labeled as groups T1, T2, T3, T4, and T5, respectively (T0: tap water treatment, control group, T1: 3.125 + 12.5 mg/L, T2: 6.25 + 25 mg/L, T3: 12.5 + 50 mg/L, T4: 25 + 100 mg/L, T5: 50 + 200 mg/L). Results shown are means ± SE (n = 3). The Tukey’s test of one-way ANOVA was used to analyze the differences in enzyme activity or gene expression levels among different groups of the same generation. Different lowercase letters indicate significant differences at the *p* < 0.05 level.

Under cadmium-and-lead treatment, the soluble trehalase (TRE1) activity of the F1 aphids was significantly increased compared to the control group (*F*_5, 12_ = 5.307; *p* = 0.008). The TRE1 activity of the F2 and F3 aphids was not significantly different compared to the control group (F2: *F*_5, 12_ = 0.976; *p* = 0.471; F3: *F*_5, 12_ = 1.348; *p* = 0.310). For the F4 and F5 aphids, the TRE1 activity was higher than that in the control group (F4: *F*_5, 12_ = 4.081; *p* = 0.021; F5: *F*_5, 12_ = 4.324; *p* = 0.018) (Figure 4A). The membrane-bound trehalase (TRE2) activity of the T4 groups in F1 significantly decreased (*F*_5, 12_ = 5.953; *p* = 0.005). For the F3 and F5 aphids, the TRE2 activity in the T1 groups was higher than that in the control group (Figure 4B). The above findings suggested that ingesting cadmium-and-lead-contaminated broad beans can have an impact on the trehalose metabolism of aphids, and there was no obvious rule among different generations of aphids.

According to the qRT-PCR results, compared with the control group, the trehalase (*TRE*) expression levels of the F1 and F4 aphids were notably higher (F1: *F*_5, 12_ = 16.635; F4: *F*_5, 12_ = 9.898; *p* < 0.001). Conversely, the expression of the *TRE* gene significantly decreased in the F2 and F3 aphids (F2: *F*_5, 12_ = 22.702; F3: *F*_5, 12_ = 14.312; *p* < 0.001). There was no significant difference in the *TRE* expression level of the F5 aphids fed on cadmium-and-lead-contaminated broad beans compared to the control group (*F*_5, 12_ = 2.988; *p* = 0.056) (Figure 4C). Similarly, the expression of the trehalose-6-phosphate synthase (*TPS*) gene increased only in the F1 aphids, with the highest expression observed in the T1 treatment group (*F*_5, 12_ = 5.624; *p* = 0.007). For the F2–F5 aphids, the expression of the *TPS* gene in all treatment groups was significantly lower than that in the T0 treatment group (F2: *F*_5, 12_ = 13.102; F3: *F*_5, 12_ = 10.224; F4: *F*_5, 12_ = 8.921; F5: *F*_5, 12_ = 32.132; *p* < 0.001) (Figure 4D). Overall, these findings demonstrate distinct alterations in the relative expression levels of *TRE* and *TPS* following treatment with a Cd^2+^–Pb^2+^ mixed solution compared to the T0 treatment group.

### 2.4. Effects of Cd–Pb Treatment on Five Generations of Aphids’ Fertility

**Figure 5:**
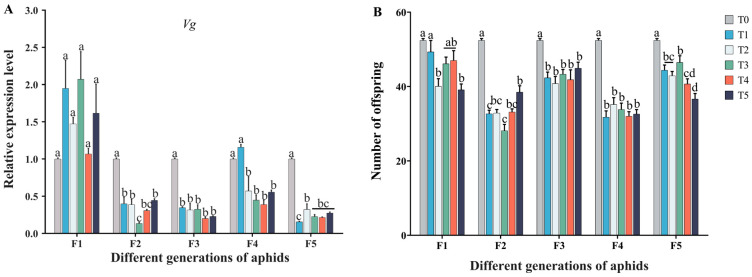
Effects of Cd–Pb treatment on five generations of aphids’ fertility. (**A**) The expression level of vitellogenin (*Vg*) gene in the adult aphids, (**B**) the total number of offspring produced by a single adult aphid from the first filial generation to the fifth filial generation within 7 days. Configured solutions of Cd^2+^ with concentrations of 3.125, 6.25, 12.5, 25, and 50 mg/L and Pb^2+^ with concentrations of 12.5, 25, 50, 100, and 200 mg/L were mixed in a 1:1 ratio. These mixtures were labeled as groups T1, T2, T3, T4, and T5, respectively (T0: tap water treatment, control group, T1: 3.125 + 12.5 mg/L, T2: 6.25 + 25 mg/L, T3: 12.5 + 50 mg/L, T4: 25 + 100 mg/L, T5: 50 + 200 mg/L). Results shown are means ± SE (n = 3). Tukey’s test of one-way ANOVA was used to analyze the differences in aphid production or gene expression levels among different groups of the same generation. Different lowercase letters indicate significant differences at the *p* < 0.05 level.

Compared with the control group, the primary precursor of egg yolk proteins, the vitellogenin (*Vg*) expression levels of the F1 aphids in the T1–T5 groups showed no significant change (*F*_5, 12_ = 2.627; *p* = 0.079). However, the expression level of the F2–F4 aphids significantly decreased (*F*_concentration,5,29_ = 10.65, *p* < 0.001) (Figure 5A). In addition, for the F1 aphids, the number of offspring produced by aphids in the T2 and T5 groups significantly decreased compared to the control group (*F*_generation,4,29_ = 81.75, *p* < 0.001). In particular, the number of offspring in the F2–F5 aphids was significantly reduced in the control group (*F*_generation,5,29_ = 48.75, *p* < 0.001) (Figure 5B). This demonstrates that exposure to the Cd^2+^–Pb^2+^ mixed solution affects the fertility of female aphids (*F*_concentration,5,29_ = 57.83, *p* < 0.001), resulting in an inhibition phenomenon. Through the results of two-way ANOVA, it was found that there was interaction between heavy metal concentration and aphid generation and it significantly affected the number of aphids’ offspring and the expression of the aphid *Vg* gene. These findings underscore that ingesting cadmium-and-lead-contaminated broad beans can profoundly inhibit aphid fecundity and impact the expression of genes related to reproduction.

### 2.5. Effects of Cd–Pb Treatment on Ladybug Developmental Duration and Weight

**Figure 6:**
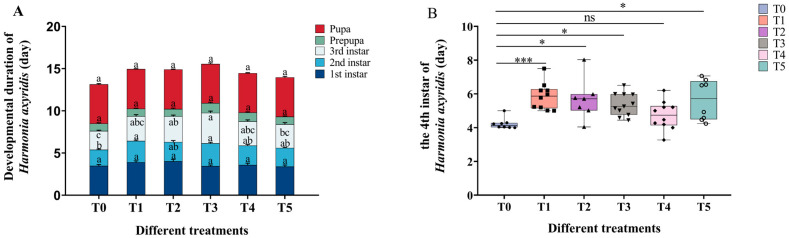
Effect of Cd–Pb stress on the developmental duration of *Harmonia axyridis*. (**A**) One-way ANOVA was used to analyze the developmental duration of *H. axyridis*, (**B**) Kruskal–Wallis test was used to analyze the fourth instar of *H. axyridis.* Configured solutions of Cd^2+^ with concentrations of 3.125, 6.25, 12.5, 25, and 50 mg/L and Pb^2+^ with concentrations of 12.5, 25, 50, 100, and 200 mg/L were mixed in a 1:1 ratio. These mixtures were labeled as groups T1, T2, T3, T4, and T5, respectively (T0: tap water treatment, control group, T1: 3.125 + 12.5 mg/L, T2: 6.25 + 25 mg/L, T3: 12.5 + 50 mg/L, T4: 25 + 100 mg/L, T5: 50 + 200 mg/L). Results were expressed as the mean ± SE. Kruskal–Wallis test was used to analyze the differences between different treatments at the fourth instar. The results are represented by * (*** *p* < 0.001; * *p* < 0.05; ns: no significant). Tukey’s test was used to analyze the differences between different treatments at the same developmental stage. Different lowercase letters indicate significant differences at the *p* < 0.05 level.

Although cadmium-and-lead treatment did not affect the development duration of the prepupal to the pupal stages (*F*_5, 48_ = 0.067, *p* = 0.997), the duration from the second instar to the third instar was significantly longer in the all the treatment groups compared to the T0 treatment group (second instar: *F*_5, 55_ = 4.514, *p* = 0.002; third instar: *F*_5, 55_ = 1.852, *p* < 0.001) (Figure 6A). The analysis of the Kruskal–Wallis test showed that there was no significant change between the T4 treatment group and the control group (*p* > 0.05) (Figure 6B). When ladybugs fed on cadmium-and-lead-treated aphids, the weight of the ladybugs at the second instar was not significantly different from that of the control group *(F*_5, 55_ = 0.438, *p* = 0.820). However, the weight of the third instar development to adult stage was significantly lower than that of the T0 treatment group from prepupal development to pupal stage (*p* < 0.01) (Table 1). It can be observed that, after herbivorous insects feed on plants treated with heavy metals, the heavy metals can be transferred along the food chain to the bodies of predatory insects, affecting the developmental period and weight of the insects.

## 3. Discussion

Heavy metals can be transferred through the food chain and accumulate in various trophic levels. The results of this study indicate that there are significant differences in the cadmium and lead contents in aphids and ladybugs due to the different concentrations of Cd^2+^ and Pb^2+^ transmitted through the food chain (Figure 1) and the accumulation of Cd^2+^ in aphids is positively correlated with the concentration of heavy metal treatment (Figure 1A). Similar results were obtained in other studies that constructed the transfer of heavy metals along the food chain of soil–plant–phytophagous insects–natural enemy insects [11,22], which further confirmed that, after heavy metal contaminated soil, it not only accumulates in phytophagous insects but also exists in related natural enemy insects along the food chain. Nevertheless, Butt et al. found that, after feeding on plants contaminated with heavy metals, *Sitobion avenae* did not accumulate Cd in its body [27]. The accumulation of heavy metals in insects is influenced by various factors such as age, gender, physiology, and genetics [28]. Our study only investigated the treatment of different batches of aphids with Cd^2+^–Pb^2+^. Therefore, further research is needed to delve more deeply into the enrichment of heavy metals along the food chain in herbivorous insects, such as different developmental stages or different genotypes. It is noteworthy that there is a positive correlation between the accumulation of cadmium in aphids and the treatment concentration. However, the change in lead content in aphids was not related to the treatment concentration (Figure 1B). There is a reference for the previous studies [29] in which the cadmium content in broad bean seedlings was correlated with the treatment concentration but not the lead content. This may be related to the extent of Cd^2+^ accumulation in plants and the detoxification ability of aphids towards Cd^2+^. Metals can be accumulated in the cytoplasm, with the amount depending on the plant species and metal. Soluble molecules in the cytoplasm, like phytochelatins (PCs), form complexes with metals and may also function as shuttles to facilitate metal transport over the tonoplast into the vacuole, such as when the PCCd complex enters the vacuole and the metal is released from the PC, which is then returned to the cytoplasm and the metal may become bound to an organic acid in the vacuole [30]. It is speculated that it is related to the detoxification mechanism of insects, for example, aphids can discharge honey dew [31]. However, the mechanism that affects the accumulation of different heavy metals in aphids still needs further exploration.

Heavy metals could be transferred to organisms along the food chain. Although organisms have a certain self-regulation function for heavy metal invasion, they may accumulate in the body beyond a certain threshold, thus inhibiting the development period and fecundity of organisms [9,32]. Our research demonstrated that the developmental period of each treatment group was generally longer than that of the control group following the administration of a cadmium–lead mixed solution (Figure 6). Numerous studies have demonstrated that insect populations experience significant suppression in response to heavy metal stress [33,34,35]. Additionally, some studies have indicated that heavy metals can reduce the gap between mitochondrial ridges in organisms, thereby inhibiting ATP synthesis and oxidative phosphorylation pathways, which in turn impedes insect development [36]. Heavy metal stress not only affects the developmental duration of insects but also inhibits their reproductive ability and has been demonstrated to exert toxic effects on eggs [37]. Vitellogenin is a critical precursor protein of egg yolk vitellin (Vn) that serves as an energy reserve in many oviparous species [38]. In this study, the relative expression level of the *Vg* gene was observed to decrease in the F2 to F5 adult aphids following their consumption of Cd^2+^–Pb^2+^-contaminated broad bean seedlings (Figure 5A). It was also proved that the influence of heavy metals on aphids’ reproduction would have a cumulative effect through many generations, especially on the expression of *Vg* genes. Shu et al. speculated that, after the adults of *Spodoptera litura* were inhibited by heavy metals, oxidative stress was induced and the nervous system of insects was damaged, which in turn disrupted the endocrine system and affected hormone secretion. Finally, the synthesis and secretion of Vg were inhibited [39]. It was also found that the intake of heavy metal-contaminated food by insects can lead to a lack of energy in the body, which may lead to a decrease in Vg synthesis, thereby inhibiting the decline in the fertility of female insects [40,41,42]. Additionally, a significant reduction in the number of offspring produced by the aphids was also noted. Heavy metals can impede the synthesis of vitellogenin peptides, thereby delaying ovary maturation, inhibiting vitellogenesis, and affecting reproductive capacity [41]. Furthermore, research has demonstrated that exposure of insects to zinc, lead, or cadmium significantly inhibits the expression of the *Vg* gene in their bodies [43]. These findings are in accordance with the results of our study, indicating that heavy metals inhibit the expression of genes associated with ovarian development, thereby affecting the reproductive capacity of insects. Zhou et al. pointed out that, under the stress of heavy metal Pb (12.5–50 mg Pb/kg in larval artificial diets), the reproductive capacity of *S. litura* was significantly affected but the *Vg* promoter was not affected. In our study, the relative expression level of the *Vg* gene in the F1 adult aphids showed no significantly different change. But in the T2 and T5 treatment groups, the number of offspring produced by aphids decreased. Whether it is related to the promoter that regulates the *Vg* gene needs further analysis [44]. Some scholars posit that insects may attempt to resist heavy metal stress by accelerating their metabolic rate, which could result in excessive energy consumption and subsequent effects on their reproductive capacity [45]. It is also noteworthy that the fecundity of predatory natural enemy insects may be affected following their feeding on heavy metal-contaminated insects [24]. However, we did not determine the expression level of the *Vg* gene in *H. axyridis* fed on aphids after heavy metal treatment. This part of the study can be added in future studies to clarify the effect of heavy metals on higher trophic organisms through food chain transmission.

It has been demonstrated that insects typically respond to heavy metal stress by reducing energy storage and accelerating the metabolic rate [46,47]. The findings of this study indicate that combined Cd^2+^ and Pb^2+^ stress led to an increase in glycogen content and glucose content in the F3 aphids (Figure 3A,B). This may be because aphids are stressed by heavy metals and the carbohydrate metabolism pathway will be affected to some extent [48,49]. Trehalose has been demonstrated to induce stress metabolic responses in insects subjected to stress, thereby mitigating damage [50]. In the present study, the activity of TRE1 and TRE2 in the F1 aphids was found to significantly decrease (Figure 4A,B) while the expression of *TPS* and *TRE* increased (Figure 4C,D), accompanied by an increase in trehalose and glucose content (Figure 3C). However, this phenomenon has not been observed in other generations, so the effect of heavy metal stress on carbohydrate metabolism of insects in many generations needs to be explored. Furthermore, Yu et al. demonstrated in their research that the *Aedes albopictus* synthesizes trehalose in response to heavy metal stress [51]. However, the results of this study also yielded contrasting findings. After feeding on zinc-contaminated rice, the expression of *TPS* genes in *Nilaparvata lugens* was found to significantly decrease [52]. Consequently, further research is required to elucidate the mechanism underlying the response of the trehalose metabolism pathway in insects subjected to continuous generations under heavy metal stress.

It can be clearly seen from the above discussion that heavy metal pollution has a profound impact on agricultural ecosystems. Heavy metals gradually accumulate in agricultural products. Once these contaminated agricultural products enter the human body through the food chain, they pose a potential serious threat to human health. In order to maintain the stability of agricultural ecosystems and human health, we must take effective measures to reduce the emission and accumulation of heavy metals. At the same time, we should also actively carry out soil remediation and ecological restoration work.

## 4. Materials and Methods

### 4.1. Insect Rearing

The aphids (*M. crassicauda*) and ladybugs (*H. axyridis*) utilized in this study were sourced from the Key Laboratory of Animal Adaptation and Evolution, School of Life and Environmental Science, Hangzhou Normal University (Hangzhou, China). Broad bean seedlings were cultivated to serve as food for the aphids. The aphids and ladybugs were reared separately in artificial climate chambers with 14 h of light and 10 h of darkness. The temperature was 19 ± 1 °C for the aphids and 25 ± 1 °C for the ladybugs. The humidity level was 70 ± 5%.

### 4.2. Experimental Design

The Risk Control Standard of Farmland Soil Pollution shows that cadmium content in soil of 0.3~3.0 mg/kg is identified as a possible risk of heavy metal pollution and higher than 3.0 mg/kg is identified as high risk and the lead content in soil of 80~400 mg/kg is identified as a possible risk of heavy metal pollution and higher than 400 mg/kg is identified as high risk [53]. At the same time, we also referred to the research of Wang et al. and Naikoo et al. to determine the experimental concentration of heavy metals in this study [22,31]. The concentrations of cadmium of 3.125, 6.25, 12.5, 25, and 50 mg/L and lead with concentrations of 12.5, 25, 50, 100, and 200 mg/L were mixed in a 1:1 ratio. These mixtures were labeled as groups T1, T2, T3, T4, and T5, respectively. This experiment used tap water treatment as control (T0). Cadmium in the form of cadmium chloride (CdCl_2_) was dissolved in tap water to make different concentrations of Cd^2+^ solution, and lead in the form of lead nitrate (Pb(NO_3_)_2_) was dissolved in tap water to make different concentrations of Pb^2+^ solution. To prevent the formation of Pb(OH)_2_, nitric acid was added to make the solution acidic (pH = 6.0). Broad bean seeds were soaked in mixed solutions of cadmium and lead at different concentrations for 24 h and then planted in the soil (the volume of nutrient soil: vermiculite: perlite = 12:4:2). Then, broad beans were irrigated with 400 mL of the cadmium-and-lead mixed solution corresponding to each concentration every three days to support normal growth.

On the 10th day after planting, 80 adult aphids (aphids that had fed on tap water-soaked and watered broad beans) were introduced to the broad bean seedlings (with irrigated mixed solutions of cadmium and lead). Through the observation of aphid development cycle, the first filial generation (F1) aphids were collected on the 10th day after transfer. That is, adult aphids were collected on the 20th day after broad bean planting. Simultaneously, about 80 adult aphids were transferred to new broad bean seedlings to rear the second generation of aphids. This process of aphid transfer and collection was continued to obtain adult aphids of the second, third, fourth, and fifth filial generations (F2–F5).

### 4.3. Determination of Heavy Metal Contents in Aphids and Ladybugs’ Pupae

The nearly 100 adult aphids (aphids that had fed on tap water-soaked and watered broad beans) were introduced to broad bean seedlings, and all the aphids bred by the aphids within 25 days of broad bean planting were named the first batch of aphids (P1). The second batch of aphids (P2) refers to all breeding aphids of the F1 aphids on the broad bean seedlings treated with the corresponding concentration of Cd–Pb mix solutions until the 25th day of planting the broad bean into the soil. This process of aphid transfer and collection was continued to obtain the third (P3), fourth (P4), and fifth (P5) batches of adult aphids. We opted to transfer ladybug larvae on the first day of third instar to broad bean seedlings with aphids. On the first day of pupal stage, about 30 ladybugs were collected per sample.

Inductively coupled plasma mass spectrometry (ICP-MS) (PerkinElmer NexION 2000 (MS)) was used to detect the heavy metal content of aphid and ladybug pupa. Then, 0.50 g of samples were weighed and placed in a digestion tank. Then, the samples were placed in 4 mL of nitric acid, pre-oxidized at 80 °C for 1h, and further digested using a microwave digester. Subsequently, they were transferred to a 50 mL volumetric flask with primary water for a constant volume and analyzed using ICP-MS. Three repetitions were performed for each sample (n = 3).

### 4.4. Determination of Carbohydrate Content and Trehalase Activity in Aphids

Trehalose (TRE), often termed the “blood sugar” of insects, is abundant in insect hemolymph [54,55]. It serves not only as an energy source but also aids insects in combating unfavorable environmental stressors [56,57]. Therefore, we determined the carbohydrate content and TRE activity in aphids. Thirty adult aphids (each generation, from F1 to F5) were placed in a 1.5 mL centrifuge tube. The anthrone method was used to detect the trehalose content. It was conducted according to the earlier method with some modifications [58,59]. The specific experimental procedures refer to the research conducted by Zhang [60]. Additionally, a Glucose (GO) Assay Kit (GAGO20, Sigma, St. Louis, MO, USA) was used to detect the glucose and glycogen content, along with the activity of soluble trehalase (TRE1) and membrane-bound trehalase (TRE2). A BCA Protein Assay Kit (P0012, Beyotime, Shanghai, China) was used to measure the protein content. Three repetitions were performed for each treatment (n = 3).

### 4.5. Effects of Cadmium–Lead Treatment on Aphid Fecundity

Eight broad bean seedlings were numbered from 1 to 8. In each generation of adult aphids (from F1 to F5), two adult aphids were selected and transferred to each broad bean seedling. The number of offspring produced by the aphids within 24 h was recorded, and the nymphs were then removed. The reproductive rate of a single adult aphid within 7 days was determined. Eight biological replicates were conducted for each experiment (n = 8). The aphid generations were parthenogenic generations.

### 4.6. Ladybug Developmental Duration, Weight

The eggs of the ladybugs were placed on moist paper towels, and the hatched larvae were transferred to rearing containers (plastic boxes, 3.7 cm × 3 cm × 3.3 cm). Each treatment group consisted of 15 ladybug larvae. Single larvae were reared in a rearing box. A total of 150 aphids, which fed on broad bean seedlings treated with a mixed solution of heavy metals cadmium and lead, were used to feed the ladybug larvae. The development of the ladybugs was observed daily. The molting time of the ladybugs was recorded, and a 1/10,000 balance (AL204, Mettler-toledo, Zurich, Switzerland) was used to measure ladybugs’ weight within 2 h. These data were used to calculate the developmental time and weight changes for each larval stage.

### 4.7. Real-Time Fluorescence Quantitative PCR (qRT-PCR)

Total aphid RNA was extracted using the Trizol method across each generation from F1 to F5. The purity and concentration of the RNA samples were assessed using a NanoDropTM 2000 spectrophotometer (Waltham, MA, USA). Subsequently, cDNA synthesis was performed using the PrimeScriptTM RT Reagent Kit with gDNA Eraser (Takara, Kyoto, Japan). For quantitative real-time PCR (qRT-PCR) analysis of gene expression, the TaKaRa TB Green^®^ Premix Ex TaqTM system was employed. Each reaction mixture included 2 µL of cDNA, 10 µL of TB Green, 1 µL of forward primer, 1 µL of reverse primer, and 6 µL of ddH_2_O. The qRT-PCR protocol consisted of initial denaturation at 95 °C for 30 s, followed by 40 cycles of 95 °C for 5 s and 60 °C for 20 s. The primer sequences of three genes were shown as *TRE* (5′-TGGCAAGATACTACGCACCA-3′, 5′-ATCAGCCAATACCCCACGAT-3′), *TPS* (5′-CGTGGACAGGCTAGACTACA-3′, 5′-CAGCTCAGTCTCGTCCTTGA-3′), and *Vg* (5′-GCATTAGCCACTATGTTTCA-3′, 5′-CGTATTGCTCCATTGTTGT-3′). *β-Actin* was used as reference gene (5′-GATCATTGCCCCACCAGAAC-3′, 5′-TTTACGGTGGACAATGCCTG-3′). The qRT-PCR data were analyzed using the 2^−ΔΔCT^ method [61].

### 4.8. Statistical Analysis

Tukey’s test of one-way ANOVA was performed to test the significance of differences among treatments. The data were analyzed using IBM SPSS statistics 20 software. Results were expressed as the mean ± standard error (SE). Statistical significance was defined as *p* < 0.05. A general linear model was also used to analyze the effects of aphid generation and heavy mental concentration on the aphid fecundity and relative transcript levels of *Vg*, resulting in a two-way interaction with “generation” x “heavy metal concentration”. Kruskal–Wallis analysis method was used to analyze the significance of the fourth instar development time of *H. axyridis*. All figures and tables were produced using Office 2021 and GraphPad 8.4.0 software. We then performed a linear regression analysis to assess cadmium–lead content in aphids and the heavy metal content in pupae of *H. axyridis* (R function ’glm’ R library ’emmeans’; Lenth, 2023).

## 5. Conclusions

In conclusion, it can be stated that heavy metals, such as cadmium and lead, can be transmitted and accumulated in aphids and ladybugs along the food chain. Aphids were observed to regulate trehalose metabolism in response to combined stress from Cd and Pb. Furthermore, the transmission of heavy metals along the food chain was observed to affect the fecundity of herbivorous insects as well as inhibit the development period and body weight of their natural enemies. This suggests that the population growth of both herbivorous and natural enemy insects is negatively impacted when they feed on prey that has been contaminated by heavy metals. The results presented here provide a foundation for further investigation into the biochemical metabolic changes in herbivorous and predatory insects in response to heavy metal stress. However, additional experimental analysis is necessary to elucidate the underlying causes and to gain a more comprehensive understanding of the mechanisms by which insects respond to heavy metal stress.

## Figures and Tables

**Table 1:** Weight of different groups of *Harmonia axyridis* at different instars.

Developmental Stage	Weight (mg)						
	T0	T1	T2	T3	T4	T5	Statistics
2nd instar	1.42 ± 0.25 a	1.42 ± 0.12 a	1.40 ± 0.16 a	1.23 ± 0.07 a	1.35 ± 0.09 a	1.31 ± 0.08 a	*F*_5, 55 =_ 0.438, *p* = 0.820
3rd instar	4.11 ± 0.20 a	3.56 ± 0.23 ab	3.11 ± 0.13 b	3.33 ± 0.22 ab	3.95 ± 0.14 a	3.40 ± 0.19 ab	*F*_5, 55 =_ 3.938, *p* = 0.004
4th instar	11.71 ± 0.49 ab	9.74 ± 0.48 b	9.49 ± 0.72 b	10.96 ± 0.64 ab	12.80 ± 0.65 a	10.50 ± 0.49 ab	*F*_5, 54 =_ 6.078, *p* < 0.001
Prepupa	34.26 ± 1.28 a	27.81 ± 1.27 bc	25.63 ± 1.48 c	27.05 ± 1.13 bc	31.93 ± 1.27 ab	29.40 ± 1.48 abc	*F*_5, 48 =_ 5.704, *p* < 0.001
Pupa	32.46 ± 1.22 a	25.50 ± 1.19 bc	23.77 ± 1.30 c	25.05 ± 1.03 bc	29.89 ± 1.17 ab	27.79 ± 1.40 abc	*F*_5, 48 =_ 6.942, *p* < 0.001
Adult	27.91 ± 1.11 a	21.61 ± 1.08 bc	20.26 ± 1.17 c	21.65 ± 0.99 bc	25.72 ± 1.01 ab	23.89 ± 1.07 abc	*F*_5, 50 =_ 7.568, *p* < 0.001

Note: Weight of different groups of *H. axyridis* at different instars. Configured solutions of Cd^2+^ with concentrations of 3.125, 6.25, 12.5, 25, and 50 mg/L and Pb^2+^ with concentrations of 12.5, 25, 50, 100, and 200 mg/L were mixed in a 1:1 ratio. These mixtures were labeled as groups T1, T2, T3, T4, and T5, respectively (T0: tap water treatment, control group, T1: 3.125 + 12.5 mg/L, T2: 6.25 + 25 mg/L, T3: 12.5 + 50 mg/L, T4: 25 + 100 mg/L, T5: 50 + 200 mg/L). Results are expressed as the mean ± SE (n = 3). Tukey’s test of one-way ANOVA was used to analyze the differences between different treatments at the different instars. Different lowercase letters indicate significant differences at the *p* < 0.05 level.

## Data Availability

The data presented in this study are available upon request from the corresponding author [B.T.].
